# Forecasting of Glucose Levels and Hypoglycemic Events: Head-to-Head Comparison of Linear and Nonlinear Data-Driven Algorithms Based on Continuous Glucose Monitoring Data Only

**DOI:** 10.3390/s21051647

**Published:** 2021-02-27

**Authors:** Francesco Prendin, Simone Del Favero, Martina Vettoretti, Giovanni Sparacino, Andrea Facchinetti

**Affiliations:** Department of Information Engineering, University of Padova, 35131 Padova, Italy; prendinf@dei.unipd.it (F.P.); vettore1@dei.unipd.it (M.V.); gianni@dei.unipd.it (G.S.); facchine@dei.unipd.it (A.F.)

**Keywords:** glucose sensor, time series, signal processing, data-driven modeling

## Abstract

In type 1 diabetes management, the availability of algorithms capable of accurately forecasting future blood glucose (BG) concentrations and hypoglycemic episodes could enable proactive therapeutic actions, e.g., the consumption of carbohydrates to mitigate, or even avoid, an impending critical event. The only input of this kind of algorithm is often continuous glucose monitoring (CGM) sensor data, because other signals (such as injected insulin, ingested carbs, and physical activity) are frequently unavailable. Several predictive algorithms fed by CGM data only have been proposed in the literature, but they were assessed using datasets originated by different experimental protocols, making a comparison of their relative merits difficult. The aim of the present work was to perform a head-to-head comparison of thirty different linear and nonlinear predictive algorithms using the same dataset, given by 124 CGM traces collected over 10 days with the newest Dexcom G6 sensor available on the market and considering a 30-min prediction horizon. We considered the state-of-the art methods, investigating, in particular, linear black-box methods (autoregressive; autoregressive moving-average; and autoregressive integrated moving-average, ARIMA) and nonlinear machine-learning methods (support vector regression, SVR; regression random forest; feed-forward neural network, fNN; and long short-term memory neural network). For each method, the prediction accuracy and hypoglycemia detection capabilities were assessed using either population or individualized model parameters. As far as prediction accuracy is concerned, the results show that the best linear algorithm (individualized ARIMA) provides accuracy comparable to that of the best nonlinear algorithm (individualized fNN), with root mean square errors of 22.15 and 21.52 mg/dL, respectively. As far as hypoglycemia detection is concerned, the best linear algorithm (individualized ARIMA) provided precision = 64%, recall = 82%, and one false alarm/day, comparable to the best nonlinear technique (population SVR): precision = 63%, recall = 69%, and 0.5 false alarms/day. In general, the head-to-head comparison of the thirty algorithms fed by CGM data only made using a wide dataset shows that individualized linear models are more effective than population ones, while no significant advantages seem to emerge when employing nonlinear methodologies.

## 1. Introduction

Type 1 diabetes (T1D) is a metabolic disease characterized by an autoimmune destruction of the pancreatic cells responsible for insulin production and thus compromises the complex physiological feedback systems regulating blood glucose (BG) homeostasis. As a consequence, T1D people are requested to keep their glycemia within a safe range (i.e., BG of 70–180 mg/dL). In particular, concentrations below or above this range (called hypo- and hyperglycemia, respectively) can represent a risk to the patient’s health, with the possibility of causing severe long-term complications.

The management of T1D therapy, which is mainly based on exogenous insulin infusions, requires the frequent monitoring of BG concentrations. Today, such monitoring is performed using continuous glucose monitoring (CGM) sensors, which allow collecting and visualizing glucose concentrations almost continuously (e.g., every 5 min) for several days [1,2]. All commercial CGM devices are labeled as minimally invasive since they require either a microneedle or a small capsule to be inserted in the subcutis, and they represent an important innovation because they allow reducing the burden of performing multiple daily invasive self-monitoring tests of BG concentrations. Of note, in recent years, there has been a great effort in investigating noninvasive glucose monitoring technologies (see [3,4,5,6] for reviews on the topic). Noninvasive CGM devices represent a further step in reducing the burden related to the daily management of T1D, but unfortunately, they are all still prototypes.

CGM devices have proved to be useful in improving insulin therapy and, in general, T1D management [7,8,9], and they are currently accepted as standard tools for glucose monitoring. Most of these devices usually provide alerts that warn the subject when the CGM values exceed the normal glucose range. Furthermore, the employment of CGM to provide short-term predictions of future glucose values or to forecast forthcoming hypo-/hyperglycemic episodes could lead to a further improvement, since targeted preventive measures—such as preventive hypotreatments (fast-acting carbohydrate consumption) [10] or correction insulin boluses [11]—could be taken to reduce the occurrence and impact of these critical episodes. Therefore, the availability of an effective BG predictive algorithm becomes of primary importance for present and future standard therapies.

In the last two decades, several algorithms for the short-term prediction of future glucose levels have been developed, using both CGM data only (to mention but a few representative examples, see [12,13,14,15,16]) and CGM data plus other available information such as the amount of ingested carbohydrates (CHO), injected insulin, and physical activity (see, for example [17,18,19,20,21] ). While the use of these additional datastreams is expected to enhance prediction performance compared to algorithms based on CGM data only [20], a nonnegligible drawback is that their application in real-world scenarios requires supplementary wearable devices (e.g., insulin pumps, mobile applications, and physical activity trackers) and actions (e.g., the safe and reliable exchange of information from one device to the other, and interactions with the user). Indeed, at present, these systems are not extensively used by individuals with diabetes [22,23]. Consequently, the possibility of efficiently performing the real-time prediction of future glucose levels with CGM data only remains, at the present time, a practically valuable option. This is the reason why investigating the performance of predictive algorithms fed by CGM data only is of primary importance.

In the last 15 years, many real-time predictive algorithms based on CGM data only have been proposed in the literature [24,25,26,27,28,29]. However, it is very difficult to establish which of them is the best performing one. Indeed, the mere comparison of performance indices extracted from different published papers could be unfair or misleading, because differences in datasets, implementation, preprocessing, and evaluation can make it difficult to claim that one prediction method is the most effective. The attempts to compare state-of-the-art methods and literature contributions on the same dataset are, to the best of our knowledge, very limited. A systematic review of glucose prediction methods was proposed by Oviedo et al., in 2017 [19]. Nonetheless, the focus of [19] was on a methodological review rather than on performing a head-to-head comparison on the same dataset. A recent comparison of different prediction algorithms on the same dataset was proposed by McShinsky et al. in [30]. A difference with the present contribution is that McShinsky et al. included both CGM-only prediction methods and algorithms relying on other signals and involved a small population (12 subjects). To fill this gap and to offer a performance baseline for future work, in this paper, we present a head-to-head comparison of thirty different real-time glucose prediction algorithms fed by CGM data only on the same dataset, which consists of 124 CGM traces of 10-day duration collected with the Dexcom G6 CGM sensor. Notably, this sensor is one of the most recently marketed, and its employment in the present paper allowed us to also assess if some previous literature findings still held with more modern, accurate CGM sensors. Specifically, we tested linear black-box models (autoregressive, autoregressive moving-average, and autoregressive integrated moving-average), nonlinear machine-learning (ML) methods (support vector regression, regression random forest, and feed-forward neural network), and a deep-learning (DL) model: the long short-term memory neural network. For the linear and ML methods, we considered both population and individualized algorithms. The former are one-fits-all algorithms, designed to work on the entire population; the latter are algorithms customized for each single patient based on their previously collected data, in order to deal with the large variability in glucose profiles among individuals with diabetes. Moreover, given the different nature of glucose fluctuations during the day and night (larger in the former case due to meal ingestion and less pronounced in the latter case) [14,20], we designed specific versions for these two time periods. With regard to model training, we opportunely divided the dataset into training and test sets, also performing a Monte Carlo simulation to avoid the possibility of the numerical results being related to a specific training-test partitioning. The performance of all the algorithms was evaluated on a 30 min prediction horizon (PH) focusing on both prediction accuracy and the capability of detecting hypoglycemic events.

The results show that the prediction accuracy of the best-performing linear and nonlinear methods are comparable, while the first slightly outperforms the second in terms of hypoglycemic prediction. In general, the results support the importance of individualization, while no significant advantages emerged when employing nonlinear strategies.

## 2. The Considered Prediction Algorithms

**Figure 1:**
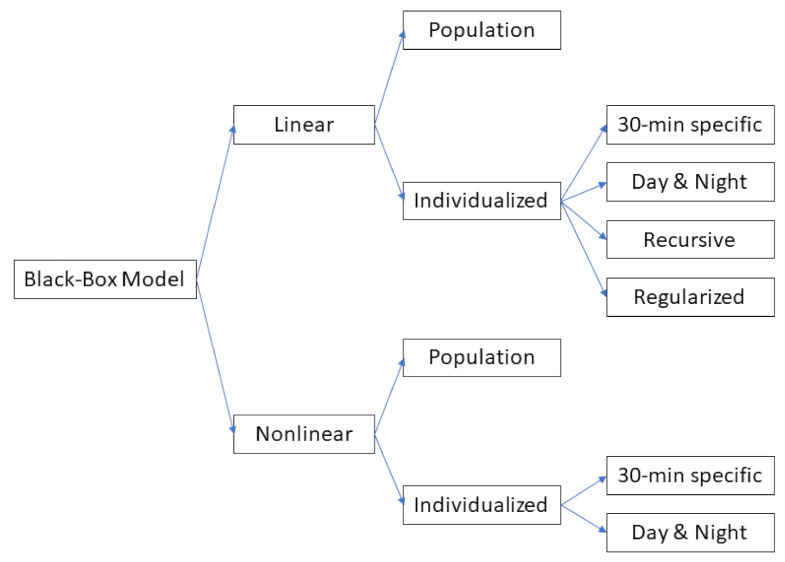
Schematic diagram of all the possibilities presented in this work.

Several options for creating the different variants of the considered classes of prediction algorithms were investigated. In order of complexity, the first option was to consider a population algorithm that computes the prediction of the future CGM value by using the same model (i.e., structure and/or order) and the same parameter value for all the individuals, i.e., without any personalization. This has the practical advantage that the model training can be performed only once, e.g., when the algorithm is designed, and the model learning procedure can leverage large datasets of CGM traces. The downside of this approach is that the prediction algorithm is not customized according to individual data [24]. Another option, with complexity higher than that of the previous one, is to develop subject-specific algorithms, which allow taking into account the large interindividual variability characterizing T1D individuals. The drawback of this approach is that the model training must be repeated for each individual in order to enable personalized glucose predictions. A further level of complexity is to consider multiple models for each individual, e.g., one for day time and one for night time. The key idea behind this choice is that the “day-time” model should be able to learn the glucose dynamics perturbed by all the external events (e.g., meals, insulin injections, and physical activity), whereas the “night-time” model should be able to learn the smoother dynamics present at night time [20]. Since no information on sleep time was available in our dataset, we decided to define day time as the interval from 6:00 up to 23:00 and night time as that from 23:05 up to 5:55. According to the rationale discussed above, the resulting categories of prediction algorithms tested in this work are summarized in the tree diagram of Figure 1. For each category, several different model classes were considered, for a total of 30 different prediction algorithms. A detailed description of the prediction algorithms tested is provided in the following two subsections.

### 2.1. Linear Black-Box Models

The linear prediction algorithms were based on a model of the CGM time series. The models were derived by using the standard pipeline described in detail in [31]. The first three steps, i.e., the choice of the model class, model complexity, and parameter estimation, were related to the model learning. The last step was model prediction, which dealt with the computation of the predicted value, starting from the model and past CGM data. These four steps are described below.

#### 2.1.1. Choice of the Model Class

Three linear model classes were considered: autoregressive (AR), autoregressive moving average (ARMA), and autoregressive integrated moving average (ARIMA) models. In the following sections, we use the notation AR(p), ARMA(p,m), and ARIMA(p,m,d), indicating with p, m, and d the order for the AR, MA, and integrated (I) part, respectively.

#### 2.1.2. Model Complexity

Once the model class was fixed, the model complexity, i.e., the number of parameters to be estimated, had to be chosen. Common techniques used for this purpose are the Akaike information criterion (AIC), the Bayesian information criterion (BIC), and cross validation (CV) [31,32]. The model orders p and m were, respectively, searched in the sets P = 1,2,…,30 and M = 0,1,…,15. After a preliminary analysis, showing that no significant differences could be seen between these methods (not shown), the BIC was chosen as the method for selecting the best model orders. Concerning the individualized linear models, we investigated a partial personalization: the model complexity of the population algorithms was maintained, but the parameter values were subject-specific (a model with individualized parameters and population orders). Then, a complete personalization was achieved by learning both the model complexity and the parameter values from patient data (a model with individualized parameters and individualized orders).

#### 2.1.3. Parameter Estimation

The first approach we used to estimate model parameters was the state-of-the-art prediction error method (PEM) [31], based on the minimization of the one-step prediction error. Furthermore, since we focused on 30-min-ahead prediction, we also considered the possibility of identifying the model parameters that minimized the 30-min-ahead prediction error (30 min-specific) rather than the 5-min-ahead error as prescribed by the standard pipeline.

With these estimation techniques, CGM time series were described by models with fixed structures and time-invariant parameters. To better follow intrapatient variability, we also investigated recursive least-squares (RLS) parameter estimation [33], which was applied, without any loss of generality, only to the AR(1) model, since previous work demonstrated the effectiveness of the AR-RLS(1) configuration [34]. Note that the RLS estimation requires setting an additional parameter, the forgetting factor, which represents a memory term for past input data [35]. This AR-RLS(1) falls into the category of a model with a fixed structure but time-varying parameters. Another option we considered was the regularized PEM approach, which considers AR models of elevated order (e.g., 100) and adds to the standard PEM cost function a regularization term representing a suitable prior on the unknown coefficients, which allows avoiding overfitting [32]. A suitable prior, known as stable spline kernel, was adopted in this work [36].

To avoid unstable models being used for the forecasting, the choice of the model complexity and the parameter-estimation steps were repeated until a stable model was identified.

#### 2.1.4. Model Prediction

Once a linear model was available from the previous steps, the k-step-ahead prediction could be derived from that model for any value of k. This was performed by applying a standard Kalman filter framework [31]. We used this approach to derive the 30-min (k = 6)-ahead prediction. We decided to focus on PH = 30 min only for two main reasons. First, the literature work [10,25], and [37] has shown that efficient corrective actions (e.g., hypotreatments or pump suspension [25,37]) triggered 20–30 min before hypoglycemia are effective in avoiding/mitigating the episodes. Second, it has been shown that PH = 30 min is a good trade-off between limiting the error of the prediction outcome (the higher the PH, the higher the error) and the effectiveness of the prediction [38].

### 2.2. Nonlinear Black-Box Models

A learning pipeline similar to that adopted for the linear models was employed for ML and DL predictive algorithms. The main steps in the learning phase were the choice of the model class, the tuning of hyperparameters (the counterpart of the model complexity), and model training (i.e., parameter estimation). The last step consisted of computing the 30-min-ahead glucose prediction once the nonlinear model was obtained.

#### 2.2.1. Choice of the Model Class

Three ML models, successfully used in a wide range of regression problems, were considered: support vector regression (SVR) [39,40], regression random forest (RegRF) [41], and feed forward neural network (fNN) [42]. In addition, we considered a DL model, namely, long short-term memory (LSTM) network, which has shown promising results in glucose prediction [43,44]. The key idea of the SVR model is to map CGM data into a higher-dimensional feature space via a nonlinear mapping and, then, to perform a linear regression in such space [45]. The goal of SVR is to find a function that has, at most, ϵ deviation from the target in the training data. Moreover, the use of adequate kernels allows dealing with linearities and nonlinearities in data [46].

RegRF is an ensemble learning method based on aggregated regression trees. A regression tree is built by recursively top–down-partitioning the feature space (composed of CGM values) into smaller sets until a stopping criterion is met. For each terminal node of the tree, a simple model (e.g., a constant model) is fitted [47]. The prediction of RegRF is obtained by combining the output of each tree.

The fNN model allows learning complex nonlinear relationships between input and output values [48]. It is composed of a set of neurons organized in layers (input, hidden, and output layers). Each neuron is characterized by a nonlinear function, e.g., sigmoid, which provides the input for the next layer, and by weights and biases. These parameters are learned from the data and are determined in order to achieve the minimum value of the cost function during the training phase. The output layer is a linear combination of the output of the previous layers.

LSTM is a useful model when maintaining long-term information over time is relevant to learn dependency and dynamics from data [49]. The key element of the LSTM model is the memory cell composed of four gates (forget, input, control, and output gates) that decide whether the information must be kept or removed from this cell at each time step. Note that, given the large number of parameters needed by LSTM and the relatively short CGM time series available for each subject in the dataset, in this work, it was not possible to apply the individualized approach for LSTM. Thus, for the LSTM model, we limited the analysis to the population approach only. In addition, since the focus of the paper is on a predictive algorithm fed by CGM data, the LSTM features were lagged CGM samples only.

A detailed review of these methods is beyond the scope of this work, and we defer the interested reader to the original work or to [50].

#### 2.2.2. Input Size and Hyperparameter Tuning

For each ML model, the optimal input size (i.e., the number of consecutive CGM readings) and other model-specific hyperparameters were chosen by using a grid search approach combined with hold-out-set CV [31] to avoid overfitting. A list of the model-specific hyperparameters and their values are reported in Table 1.

Concerning LSTM, given the dimensions of our dataset and the elevated number of hyperparameters to be tuned, we decided to manually set some of them, such as the number of layers, learning rate, and decay factor, on the basis of literature studies to avoid the risk of overfitting [44,51]. This approach proved to be efficient in reducing such a risk in even more complex and deep neural networks [15,16,21]. Moreover, to further strengthen the learning phase, we added to our LSTM a dropout layer, which randomly ignored neurons during the training. Finally, based on the results of the hold-out-set CV, we found that the optimal LSTM structure consisted of a network composed of a single LSTM layer, 30 hidden nodes, and 10 lagged CGM values as input.

As for the individualized linear models, we also investigated a partial personalization for nonlinear ones: the hyperparameters and optimal input size of the population algorithms were maintained, but the parameter values were subject specific (a model with individualized parameters and population hyperparameters). Then, a complete personalization was achieved by determining the model-specific hyperparameters, the optimal input size, and the parameters based on individual data (a model with individualized parameters and individual hyperparameters).

#### 2.2.3. Model Training

Independently of the algorithm considered (i.e., population, individualized, or day/night specific), the CGM data were standardized using z-score standardization [50]. Then, parameter estimation was performed by minimizing the model-specific loss function through the use of specific optimized versions of the stochastic gradient descent algorithm.

#### 2.2.4. Model Prediction

The three previous phases allow learning a model that can directly produce the 30-min-ahead-in-time prediction, once fed by a sequence of standardized CGM data.

## 3. Criteria and Metrics for the Assessment of the Algorithms

The algorithms were compared considering both the accuracy of the glucose value prediction and the hypoglycemia event detection capability.

### 3.1. Glucose Value Prediction

The predicted glucose profiles were evaluated with three commonly used metrics. First, we considered the root mean square error (*RMSE*) between the predicted glucose values and measured CGM data:

(1) $$\begin{array}{cc} RMSE= & \frac{1}{\sqrt{N}}\left|\right|\left(y\left(t\right)-\hat{y}\left(t|t-PH\right)\right){\left|\right|}_{2}=\sqrt{\frac{1}{N}\sum_{t=1}^{N}{\left(y\left(t\right)-\hat{y}\left(t|t-PH\right)\right)}^{2}} \end{array}$$

where *PH* is the prediction horizon, *N* is the length of the subject CGM data portion in the test set, y(t) is the current CGM value, and $\hat{y}\left(t|t-PH\right)$ is its PH-step-ahead prediction. By ${\left||x\left(t\right)|\right|}_{2}$, we denote the Euclidean norm of the signal *x(t)*, namely: ${\left||x\left(t\right)|\right|}_{2}=\sqrt{\sum_{t=1}^{N}{\left(x\left(t\right)\right)}^{2}}$.

RMSE takes positive values, with RMSE = 0 corresponding to the perfect prediction, and increasing RMSE values corresponding to larger prediction errors.

Furthermore, we also considered the coefficient of determination (COD):

(2) $$\begin{array}{c} COD=100\;\cdot \;(1-\frac{\left|\right|\left(y\left(t\right)-\hat{y}\left(t|t-PH\right)\right){\left|\right|}_{2}^{2}}{\left|\right|\left(y\left(t\right)-\bar{y}\left(t\right)\right){\left|\right|}_{2}^{2}}) \end{array}$$

where $\bar{y}$ is the mean of the CGM data. The COD presents the maximum value (i.e., 100%) if the predicted profile exactly matches the target CGM signal. If the variance of the prediction error is equal to the variance of the signal or, equivalently, if the prediction is equal to the mean of the signal, the COD is 0%. There is no lower bound for COD values (they may also be negative).

Finally, the delay existing between the CGM signal and the predicted profile is defined as the temporal shift that minimizes the square of the mean quadratic error between the target and the prediction:

(3) $$\begin{array}{c} delay=\underset{j\in \left[0,PH\right]}{arg min}\left[\frac{1}{N}\sum_{t=1}^{N-PH}{\left(\left(\hat{y}\left(t|t-PH\right)+j\right)-y\left(t\right)\right)}^{2}\right] \end{array}$$

Of course, the lower the delay, the prompter and more useful the prediction. A delay equal to the PH means that the model prediction is not better than looking at the current glucose level. Finally, in order to investigate if significant differences existed among the compared algorithms, a one-way analysis of variance (ANOVA) was used to compare the RMSE values. A significance level of 5% (*p*-value < 0.05) was considered in all cases. The adjustment for multiple comparisons was performed by using the Bonferroni correction.

### 3.2. Hypoglycemia Prediction

**Figure 2:**
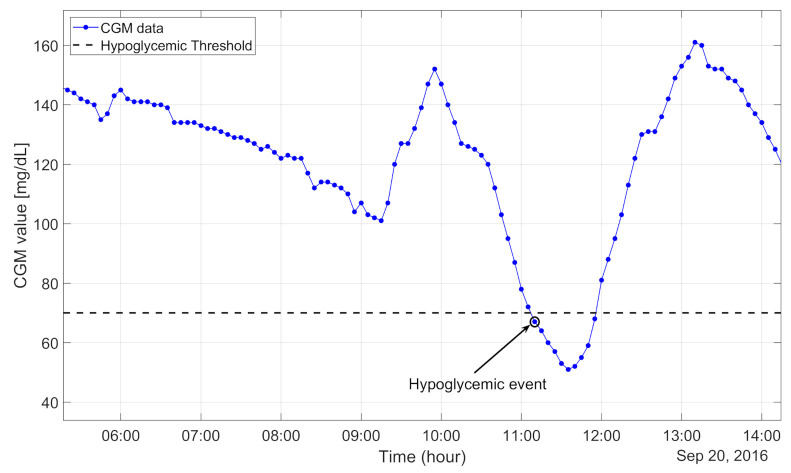
Example of real continuous glucose monitoring (CGM) data of hypoglycemic event onset.

Concerning the assessment of the ability to predict hypoglycemic events, following [38], we defined the occurrence of a new hypoglycemic event when a CGM value below 70 mg/dL was observed and the previous six CGM readings were above 70 mg/dl. An example of a hypoglycemic event is shown in Figure 2. Hypoglycemic alarms were defined for the predicted CGM signal with exactly the same criteria used for hypoglycemic event definition.

#### Hypoglycemia Prediction Metrics

**Figure 3:**
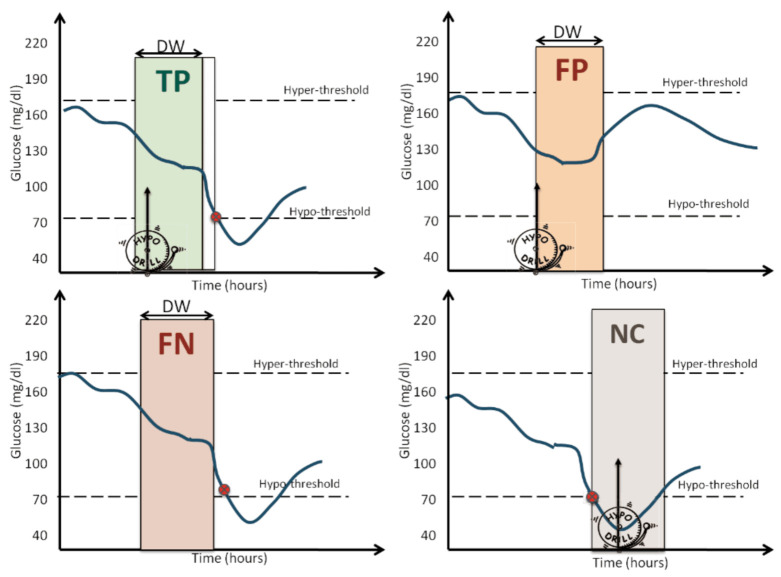
Examples of true positive (**top-left** corner), false positive (**top-right** corner), false negative (**bottom-left** corner), and not countable (**bottom-right** corner).

Considering a PH = 30 min and detection window (DW) of 40 min, we assigned:

- True positive (TP): if an alarm was raised at least 5 min before the hypoglycemic event and at most DW+5 min before that episode, as shown in Figure 3 (top left panel). According to this definition, alarms raised with a time anticipation larger than DW+5 min were not counted as TPs, because it was difficult to claim a match between the alarm and the hypoglycemic event;
- False positive (FP): if an alarm was raised, but no event occurred in the following DW minutes, as shown Figure 3 (top-right panel);
- False negative (FN): if no alarm was raised at least 5 min before the event and at most DW+5 min before the event, as shown in Figure 3 (bottom-left panel);

Finally, we defined as late alarms the alarms raised within DW minutes after the hypoglycemic event, as shown in Figure 3 (bottom-right panel). Late alarms were considered neither TPs nor FPs, i.e., the events corresponding to late alarms were not counted (NC) in the computation of the event prediction metrics. The calculation of true negatives (TNs) was of limited interest [52], since we were dealing with an unbalanced dataset (only a few hypoglycemic events in 10 days of monitoring).

Once the TPs, FPs, and FNs were found, the following metrics were used to evaluate the different models:

(4) $$precision=\frac{TP}{TP+FP}$$

(5) $$recall=\frac{TP}{TP+FN}$$

(6) $$F1-score=2\;\cdot \;\frac{precision\;\cdot \;recall}{precision+recall}$$

The precision (4) is the fraction of the correct alarms over the total number of alarms generated. The recall (5), also called the sensitivity, is the fraction of correctly detected events over the total number of events. The F1-score (6) is the harmonic mean of the two previous metrics. Since the dataset is strongly unbalanced, we also evaluated the daily number of FPs generated by the algorithm (FPs per day). We also evaluated the time gain (TG) of the hypoglycemic alert as the time between the alert and the real hypoglycemic event.

Unlike the glucose prediction metrics, for which a different metric value was calculated for each subject, the values of the hypoglycemia prediction metrics were obtained by considering all the hypoglycemic events of the different subjects, as they belonged to a unique time series.

## 4. The Dataset and Its Partitioning

The data were kindly provided by Dexcom (Dexcom Inc., San Diego, CA, USA) and taken from the pivotal study of their last commercial sensor (Dexcom G6 CGM sensor), described at ClinicalTrials.gov (NCT02880267). This was a multicenter study, involving 11 centers. Each center obtained approval from the local IRB/ethical committee, as reported in the main publication associated with the study [53]. The original dataset included 177 CGM traces collected in 141 T1D adults (aged 18+) by the Dexcom G6 sensor (36 subjects wore two sensors in parallel). For the purposes of this work, we selected 124 CGM traces, keeping only one CGM datastream for each subject and discarding subjects who wore the CGM devices for less than 10 consecutive days. The sampling time was 5 min. In summary, the dataset granted us 1240 days of CGM data, ~350,000 samples and more than 19,200 CGM samples below 70 mg/dL (i.e., 5.4% of the total samples), with ~1600 hypoglycemic episodes. It should be noted that, even though hypoglycemia was rather rare in the real data, the large dataset adopted and the consequent abundant number of hypoglycemic episodes allowed a solid assessment of the algorithm’s ability to predict a hypoglycemic episode. Moreover, the number of hypoglycemic episodes present in our dataset was significantly larger than those of other papers having the same aim [14,54]. A detailed description of the clinical study can be found in [53].

### 4.1. Training and Test Set

A comparison of the proposed prediction algorithms was obtained by evaluating the performance of each method on a same test set. A total of 20% of all the CGM traces (i.e., 25 CGM time series) were randomly chosen from the original dataset and were candidates as a test set for evaluating all the predictive algorithms. The remaining time series (i.e., 99 CGM traces) were used to train the population algorithms. Concerning the training of the individualized algorithms, the 25 CGM time series, the candidates as a test set, were split into training and test sets. In a preliminary examination, we found that the dimension of the training set should be approximately 7 days for nonlinear models. However, the linear algorithms required 33 h of CGM data for the training phase only. Therefore, the test set, identical for all the algorithms, was composed of the last 3 days (out of 10 days) of the 25 CGM time series initially chosen. By doing so, the CGM data of the training and test set were completely independent.

Since during data acquisition, failures and missed data may occur, the CGM traces, in the training set only, were preprocessed as follows: first, they were realigned to a uniform temporal grid, and if there was a data gap and it was smaller than 15 min, missed values were imputed via third-order spline interpolation. If the gap was longer than 15 min, the CGM trace was split into different segments.

### 4.2. Monte Carlo Simulations

Splitting the dataset as described in the foregoing subsection had the advantage of providing a test set that was the same for all the algorithms but had the issue that the test set was small (about 75 days over the total 1240), thus containing a limited number of hypoglycemic episodes (~90 over about 1600 total hypoglycemic events). Both the glucose and hypoglycemic prediction performance were randomly affected by the choice of the test set. In fact, one test set extraction might turn out to be particular advantageous for algorithm A and penalizing for algorithm B, while another could result in the opposite. This issue could be overcome by performing a Monte Carlo simulation: the procedure of randomly splitting the dataset into training and test sets was iterated several times (i.e., 100). For each iteration, a new training and test set was obtained, and then, the glucose prediction analysis described in this work was performed.

## 5. Results

### 5.1. Illustration of a Representative Training–Test Partitioning Example

Glucose prediction and hypoglycemic event detection performance with a representative training–test partition, chosen among the 100 Monte Carlo simulations, are shown in Table 2 for linear models and in Table 3 for nonlinear models. In particular, in Table 2 and Table 3, the glucose prediction metrics are reported as median value [interquartile range] over the 25 CGM time series used as the test set. Finally, statistical analysis of the test set of this representative training–test set extraction was performed.

#### 5.1.1. Linear Black-Box Models

**Figure 4:**
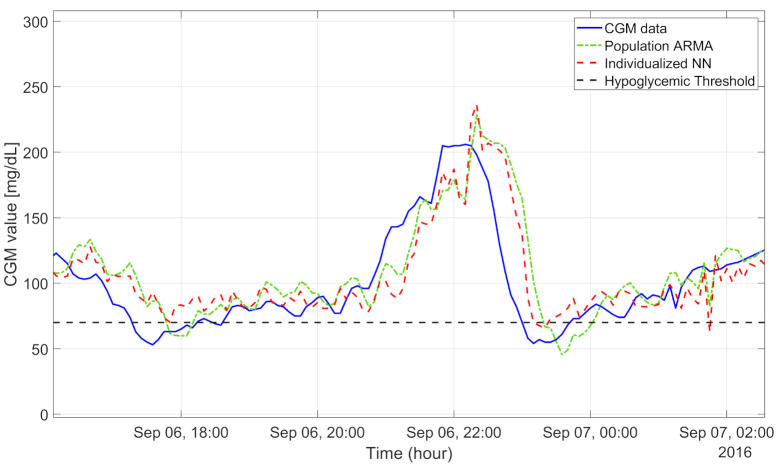
CGM data (blue line): 30-min-ahead prediction obtained with population ARMA(4,1) (green dash-dotted line) and individualized neural network (red dashed line). Hypoglycemic threshold is shown by black dashed line.

The population algorithms underestimated in hyperglycemia and overestimated in hypoglycemia, as illustrated for a representative subject in Figure 4. In particular, the CGM data (blue line) show a hypoglycemic episode before 18:00, an elevated blood glucose peak (210 mg/dL) at 22:00, and another hypoglycemic event before 00:00. In these three situations, the population ARMA(4,1) model (green dash-dotted line), for example, provided glucose prediction values quite distant from the target CGM data. In fact, the RMSEs achieved with the population ARMA and ARIMA were, respectively, about 23.75 and 23.78 mg/dL. The early detection of hypoglycemic episodes was unsatisfactory even for the population ARIMA algorithm, the best performing among the population approaches: both the precision and recall were low, respectively, at around 63% and 48%. The median TG was only 5 min.

**Figure 5:**
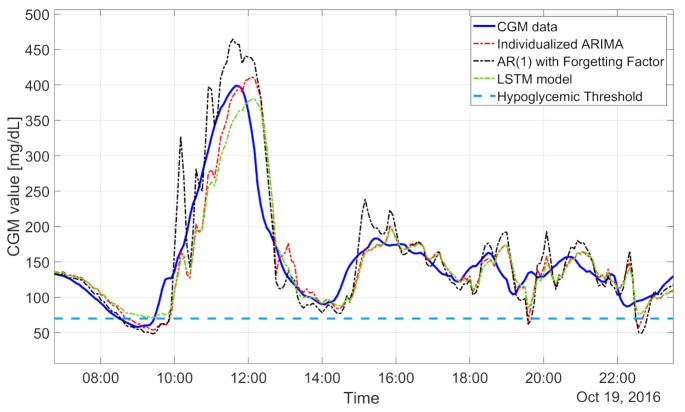
CGM data (blue line) and 30-min-ahead prediction obtained by AR-RLS(1) (black dash-dotted line), individualized ARIMA(2,1,1) (red dash-dotted line), and LSTM model (green dash-dotted line). Hypoglycemic threshold (light blue dashed line).

Looking at the results in Table 2, we can note that the individualized models outperformed the population ones: the RMSEs provided by the population AR and by the individualized AR were, respectively, around 23.63 and 22.76 mg/dL. The detection of hypoglycemic events was also increased with the AR individualized models. Indeed, the recall and precision were around 40% and 58%, respectively, with the individualized models and around 48% and 46%, respectively, with the population models. The median TG improved from 5 min with the population AR to 10 min with the individualized AR. In particular, individualized ARIMA models allow mitigating the impact of slow changes in glucose mean concentrations. Thus, the corresponding predicted profiles turned out to be more adherent to the target signal, as visible in the representative subject of Figure 5 (individualized ARIMA(2,1,1), whose prediction is reported by the red dash-dotted line, provided accurate predictions when the CGM data fell below the hypoglycemic threshold, i.e., from 8:00 to 10:00). These features make individualized ARIMA the best-performing linear algorithm both for glucose value prediction, granting a median RMSE of 22.15 mg/dL, and for hypoglycemic event prediction, with a recall of 82% and precision of 64%. One might expect that the model derived by minimizing the 30-min-ahead prediction error would achieve better performance than the model obtained following the standard PEM pipeline, i.e., by minimizing the 5-min prediction error and then deriving the predictor.

However, this is not the case, and it can be seen that the 30-min AR model provides similar performance (RMSE: 22.79 mg/dL, COD: 83.89%, recall: 21%, and precision: 42%) to the individual models identified with the standard PEM approach (RMSE: 22.76 mg/dL, COD: 84.53%, recall: 40%, and precision: 58%). This is in line with the theory in [31,32].

The day-and-night-specific algorithms provided higher RMSEs (24.22, 24.37, and 23.1 mg/dL for AR, ARMA, and ARIMA, respectively) than the algorithms described previously. The hypoglycemic detection was comparable to that with the individualized models. The extra complexity of the day-and-night-specific models does not seem to be justified by better performance. The regularized models performed very similarly to the individualized models (RMSE: 23.23 mg/dL, while the recall and precision were, respectively, 50% and 60%) but require a more complicated identification procedure. Finally, concerning AR-RLS(1), it allows rapidly tracking changes in glucose trends (Figure 5, black dash-dotted line), but it can be very sensitive to noisy CGM readings, and the resulting RMSE was higher than those for the other algorithms investigated (27.43 mg/dL). This feature was also reflected in an increased number of false alarms generated (about one/day). However, both the recall and precision were high: 86% and 55%, respectively. The median TG was 15 min. In summary, the best linear model was given by individualized ARIMA. Finally, statistically significant differences between the RMSE results obtained with the population algorithm and the results obtained by the individualized algorithm are indicated in Table 2 by asterisks.

#### 5.1.2. Nonlinear Black-Box Models

Considering the population models, the best ML method for the detection of hypoglycemic events was SVR fed by 50 min of CGM data with a Gaussian kernel, which presented TG = 10 min, recall = 69%, precision = 63%, and one false alarm every 2 days. Despite the good results in terms of event detection, it should be noted that the RMSE was around 22.85 mg/dL. The RegRF achieved the highest RMSE among the population nonlinear models considered: 23.42 mg/dL. Furthermore, we noted by visual inspection that the predicted profiles obtained by RegRF suffered from large delays, especially when the target signal was rising. Moreover, RegRF tended to overestimate in hypoglycemia, generating a recall around 20% and a precision of 36% only.

The minimum RMSE was achieved by an fNN fed by 50 min of CGM data, composed of two hidden layers, each of them with 10 neurons, similar to what is described in [42]. Despite the RMSE being the lowest among the nonlinear population methods (21.81 mg/dL), all the hypoglycemic detection metrics were not satisfactory: the recall was 27%, the precision was 39%, and the TG was 5 min. The LSTM-predicted profile (the green dash-dotted line in Figure 5) was similar to the one obtained with an fNN: it exhibited a RMSE around 23 mg/dL, recall around 26%, and precision around 46%.

Generally, the individualization of the model hyperparameters allowed reducing the RMSE, e.g., the individualized SVR and fNN with individual hyperparameters achieved median RMSEs of 22.16 and 21.52 mg/dL, respectively. In addition, the result obtained by the individualized fNN outperformed all the 30 algorithms tested in this work. However, the slight improvement in terms of the prediction of glucose values does not imply an important improvement in hypoglycemic event prediction. In fact, the best individualized ML model for hypoglycemia forecasting was the individualized SVR, whose performance was similar to that of the population SVR model: the recall was about 59% vs. 63%, the precision was 72% vs. 69%, and the median TG was 10 min in both cases (individualized vs. population, respectively). The individual fNN provided a predicted profile that tended to underestimate in hyperglycemia and overestimate in hypoglycemia as shown in Figure 4 (the prediction of the fNN with individual hyperparameters, the red dashed line, was more adherent to the target when the CGM was inside the range 80–120 mg/dL).

Individualized RegRF provided the worst performance in terms of both glucose and hypoglycemic event prediction: the RMSE was 26.16 mg/dL, the recall was 39%, and the precision was 60%. The individualized day-and-night-specific ML algorithms provided, in general, RMSEs higher (around 30 mg/dL) than those of the algorithms described previously. The ability to detect hypoglycemic events was lower than that of the individualized ML models.

It is interesting to note that all these nonlinear methods did not provide satisfactory results in terms of hypoglycemia detection. It is worth noting that no statistically significant differences between the RMSE results obtained with the individualized nonlinear algorithms with individual hyperparameters (SVR and fNN) and the individual linear ones with individual orders (AR, ARMA, and ARIMA) can be observed.

### 5.2. Monte Carlo Analysis

The results for the glucose prediction and hypoglycemic event detection performance of the 100 Monte Carlo simulations are shown in Table 4. For each metric, we report the mean and standard deviation of all the simulations. It is worth noting that the numerical results described in the foregoing subsection were confirmed by this further analysis. Finally, the statistical analysis performed for the Monte Carlo iterations shows that no significant differences between the RMSE results obtained with the best-performing nonlinear and the best-performing linear algorithms can be observed.

## 6. Discussion and Main Findings

Among the 30 glucose predictive algorithms tested in this head-to-head comparison, the linear algorithm granting the best future glucose prediction is the individualized ARIMA (median RMSE of 22.15 mg/dL). The best nonlinear algorithm is individualized fNN (median RMSE of 21.52 mg/dL). While the median RMSE of the individualized fNN is slightly smaller than the median RMSE obtained using an individualized ARIMA, the difference among the two was not found to be statistically significant. When hypoglycemic event detection is considered, individualized ARIMA achieved the best F1-score (72%), outperforming SVR (F1-score = 65%), the best nonlinear method based on this metric. All the algorithms exhibited TGs (i.e., the temporal distances between the hypoglycemic events and the predictive alarms) that spanned from 5 up to 15 min, with the best results for individualized ARIMA and SVR. The generation of preventive hypoglycemic alerts 5–15 min before the event could be clinically relevant. In fact, in the best-case scenario in which a preventive hypotreatment is ingested 15 min before the hypoglycemic episode, the rescue CHO will likely reach the blood before the hypoglycemic event, preventing or drastically mitigating it. Even a 5-min anticipation, while probably insufficient to prevent hypoglycemia, would still contribute to reducing both its duration and its amplitude. The practical benefit of taking preventive actions before hypoglycemia with TGs similar to those reported here has been shown in [25].

Two main findings are worth being highlighted. First, the individualized methods slightly outperformed their population counterparts, confirming the positive impact of model parameter individualization, which allows customizing models for each single patient and dealing with the large variability in glucose profiles among individuals with diabetes. Second, the use of advanced nonlinear techniques, substantially more complex than their linear counterparts, did not majorly benefit the prediction performance. Clearly, this last finding does not exclude that other nonlinear ML or DL techniques could change the picture (an exhaustive exploration of nonlinear techniques is practically impossible, also considering the number of new contributions constantly proposed in these fields), but proves that linear methods are still highly valuable options that offer an excellent trade-off between complexity and performance. It is worth noting that both the numerical and statistical findings of this analysis seem to be in line with most of the literature studies [14,15,16,33,38,41,42,51]. Nonetheless, we report a clear contrast with the findings in some other contributions [43,55].

**Figure 6:**
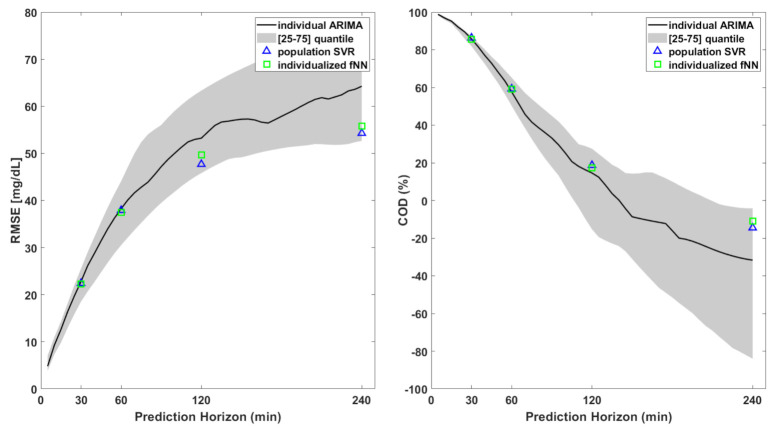
RMSE (**left**) and COD (**right**) for the 3 best-performing algorithms out of the 30 tested in this work. The black lines are the median RMSE and COD (left and right, respectively) obtained using individual ARIMA with different prediction horizons. Blue triangles and green squares indicate the same metrics for PH = 30, 60, 120, and 240 min for population SVR and individualized fNN, respectively.

All the algorithms described in this work are focused on short-term prediction (i.e., 30 min), which enables patients to take proactive/corrective measures to mitigate or to avoid critical events. As a further exploratory analysis, we evaluated the prediction performance of the best linear and nonlinear algorithms for several PHs. As shown in Figure 6, the prediction error considerably increased for long-term prediction for both the linear and nonlinear algorithms. This was expected: the larger the temporal distance, the larger the number of factors that can influence the blood glucose concentration. This further strengthens our motivation to limit the head-to-head comparison of glucose predictive algorithms fed by CGM data to only a 30 min prediction horizon.

## 7. Conclusions and Future Work

The forecasting of future glucose levels and/or hypoglycemic episodes has the potential to play a key role in improving diabetes management. Prediction algorithms using CGM data only remain, at the present time, a highly valuable option, the acquisition and synchronization of datastreams from other data sources (e.g., meal and insulin information, physical activity, etc.) not being straightforward or even impossible in a real-time setting. Several contributions in the literature have tackled this problem, but comparing their findings is not trivial due to different data collection conditions (highly controlled set-ups, such as inpatient trials, as opposed to real-life recordings), preprocessing methods, and evaluation metrics. A head-to-head comparison, removing these confounding factors, was missing. In this work, we filled this gap by systematically comparing several linear and nonlinear prediction algorithms and exploring a number of degrees of freedom in their design. Furthermore, where possible, we compared population vs. individualized prediction approaches. In total, we considered 17 algorithms based on linear black-box models and 13 based on nonlinear models. We tested all the prediction algorithms on a dataset from the Dexcom G6 CGM, one of the newest and best-performing sensors on the market. The availability of such a dataset represents an adjunctive contribution of this work, since it allows verifying if previous literature findings, often obtained with older and less-accurate sensors, remain valid on the most recent sensors on the market. The results show that individualized ARIMA and individualized fNN are the best-performing algorithms in terms of predictive performance: the median RMSEs were 22.15 and 21.52 mg/dL, respectively. When considering hypoglycemia detection, individualized ARIMA is still the best performing, and it outperformed the best nonlinear technique (population SVR), with F1-scores of 72% and 65%, respectively. In general, this head-to-head comparison of thirty algorithms fed by CGM data only made on a wide dataset shows that individualized linear models are more effective than population ones, while no significant advantages seem to emerge when employing nonlinear methodologies for a 30 min prediction horizon.

Among the limitations of this work, there is the fact that we did not consider techniques formulating hypoglycemia detection as a binary classification task. In this regard, it should be reported that a previous contribution [38], found that binary classifiers show worse performance compared to regression-based algorithms. Nonetheless, the systematic comparison of these two approaches will be the object of future work.

## Figures and Tables

**Table 1:** Nonlinear model hyperparameters.

Model	Hyperparameter	Range
SVR	Error penalty term, kernel scale factor	10${}^{-3}$–10${}^{3}$ (logarithmic scaled)
RegRF	Number of trees	10–500
	Number of leaves, max. number of splits	1-max(2,training samples) (logarithmic scaled)
fNN	Number of layers	1–3
	Number of neurons	5–20
	Activation function	Hyperbolic tangent, sigmoidal
	Max. training epochs	500–1500
fNN	Number of layers	1–3
	Number of neurons	5–20
	Activation function	Hyperbolic tangent, sigmoidal
	Max. training epochs	500–1500
LSTM	Number of hidden units	20–100
	Max. training epochs	50–1000
	Dropout rate	0.01–0.7

**Table 2:** Performance of linear algorithms with a representative dataset partitioning (30-min partition horizon (PH)). The asterisks indicate *p*-values < 0.05

Model Type			Model Class	Glucose Prediction Metric			Hypo Event Detection				
				Delay (min)	RMSE (mg/dL)	COD (%)	F1-Score (%)	Precision (%)	Recall (%)	FP/Day	TG (min)
	Population		AR *	25	23.63	80.89	47	46	48	0.41	5
				[ 23.75–25]	[20.91–32.24]	[72.39–86.59]					[5–10]
			ARMA *	25	23.75	81.23	46	45	47	0.31	5
				[20–25]	[20.75–32.15]	[72.47–86.65]					[5–10]
			ARIMA *	25	23.78	81.21	55	63	48	0.33	5
				[20–25]	[20.75–32.15]	[72.44–86.62]					[5–10]
Individual	Population order		AR	20	22.73	84.63	55	63	48	0.47	10
				[20–25]	[19.02–30.36]	[80.98–87.9]					[5–15]
			ARMA	20	22.83	84.64	51	50	52	0.85	10
				[20–25]	[19.31–30.91]	[77.01–88.36]					[5–15]
			ARIMA	25	23.12	83.36	67	64	71	0.67	10
				[20–25]	[20.22–28.65]	[78.68–87.99]					[10–15]
	Individual order		AR	25	22.76	84.53	48	58	40	0.47	10
				[20–25]	[18.76–29.47]	[80.79–88.1]					[5–10]
			ARMA *	25	22.55	83.71	36	48	29	0.51	10
				[23.75–25]	[20.16–30.46]	[76.99–87.91]					[5–15]
			ARIMA *	25	22.15	84.64	72	64	82	0.76	10
				[25–25]	[19.8–28.87]	[78.71–87.59]					[5–15]
	Individual order		AR	25	22.79	83.89	28	42	21	0.43	5
				[20–25]	[19.75–28.84]	[76.7–88.36]					[5–15]
		30 min	ARMA	25	22.89	83.37	24	39	17	0.44	5
		specific		[25–30]	[20.54–29.81]	[75.8–87.93]					[5–15]
			ARIMA *	25	22.39	84.47	64	56	75	0.57	10
				[25–25]	[19.97–29.31]	[76.28–88.23]					[5–10]
	Individual order	Day and night	AR	25	24.22	80.72	26	41	20	0.29	5
				[25–25]	[20.74–30.16]	[76.37–84.87]					[5–15]
			ARMA	25	24.37	77.31	24	39	17	0.29	10
				[25–26.25]	[21.31–30.25]	[75.49–84.72]					[5–15]
			ARIMA	25	23.1	82.2	67	70	64	0.44	10
				[25–26.25]	[20.47–29.76]	[76.95–86.74]					[5–15]
	Regularized		AR	20	23.23	82.52	54	60	50	0.55	10
				[20–25]	[19.85–31.01]	[77.22–87.74]					[5–20]
	RLS		AR	30	27.43	75.66	68	55	86	0.88	15
				[25–30]	[24.63–33.88]	[67.77–81.16]					[10–25]

**Table 3:** Performance of nonlinear algorithms with a representative dataset partitioning (30-min PH).

Model Type			Model Class	Glucose Prediction Metric			Hypo Event Detection				
				Delay (min)	RMSE (mg/dL)	COD (%)	F1-Score	Precision	Recall	FP/Day	TG (min)
	Population		SVR	25	22.85	85.14	65	63	69	0.53	10
				[25–25]	[18.81–28.61]	[79.35–88.15]					[5–15]
			RegRF	30	23.42	80.65	25	36	20	0.3	5
				[30–30]	[21.29–30.86]	[72.83–84.91]					[5–10]
			fNN	20	21.81	86.19	31	39	27	0.36	5
				[20–25]	[18.65–27.86]	[81.1–89.41]					[5–11.25]
			LSTM	25	23.1	82.31	33	46	26	0.3	5
				[20–25]	[20.26–28.75]	[77.54–87.33]					[5–10]
Individual	Population hyperparameters		SVR	25	21.97	84.22	64	72	59	0.31	10
				[25–25]	[19.68–28.98]	[78.78–87.39]					[5–15]
			RegRF	30	23.81	72.73	25	33	21	0.03	5
				[30–30]	[21.35–30.47]	[67.85–79.93]					[5–5]
			fNN	20	21.76	83.98	47	59	40	0.45	10
				[20–25]	[18.89–28.97]	[79.37–88.7]					[5–18.75]
	Individual hyperparameters		SVR	20	22.16	81.97	54	57	52	0.62	10
				[20–25]	[20.62–28.79]	[65.89–87.45]					[10–20]
			RegRF	25	26.16	77.14	47	60	39	0.42	12.5
				[25–25]	[22.49–33.97]	[69.79–82.47]					[5–20]
			fNN	20	21.52	85.37	47	57	40	0.47	10
				[20–25]	[19.12–28.29]	[78.78–88.11]					[5–18.75]
	Individual hyperparameters	Day and night	SVR	25	30.13	67.75	48	61	40	0.41	10
				[20–25]	[25.17–40.9]	[57–76.34]					[5–20]
			RegRF	25	33.34	68.47	39	53	31	0.43	10
				[25–25]	[26.84–37.71]	[62.71–74.49]					[10–20]
			fNN	20	24.4	82.11	33	53	24	0.34	10
				[20–25]	[20.88–29.89]	[74.84–86.19]					[5–17.5]

**Table 4:** Performance of nonlinear algorithms with a representative dataset partitioning (30-min PH).

Model Type			Model Class	Glucose Prediction Metric			Hypo Event Detection				
				Delay (min)	RMSE (mg/dL)	COD (%)	F1-Score	Precision	Recall	FP/Day	TG (min)
	Population		AR	25(0)	23.86(2.44)	79.8(3.18)	46.97(6.04)	54.33(6.8)	41.64(6.45)	0.48(0.12)	8.32(2.21)
			ARMA	25(0)	23.75(2.43)	79.86(3.17)	47.17(5.81)	54.77(6.8)	41.69(6.16)	0.47(0.12)	8.09(2.25)
			ARIMA	25(0)	23.96(2.42)	80.06(3.16)	50.27(5.18)	58.24(6.32)	44.51(5.7)	0.44(0.13)	9.18(1.8)
Individual	Population order		AR	21.45(2.29)	22.79(1.57)	84.83(1.82)	44.12(7.03)	51.99(6.63)	38.58(7.57)	0.48(0.1)	9.59(1.42)
			ARMA	21.55(2.33)	22.89(1.59)	84.05(1.84)	41.47(6.31)	48.38(6.03)	36.66(7.22)	0.53(0.15)	9.64(1.89)
			ARIMA	24.73(1.15)	22.74(1.8)	83.74(1.64)	62.83(5.6)	56.23(6.12)	71.67(6.8)	0.77(0.19)	11.73(2.4)
	Individual order		AR	24.45(1.57)	22.78(1.67)	84.56(1.86)	49.73(7.45)	58.77(7)	43.11(7.78)	0,48(0.11)	9.64(1.31)
			ARMA	25(0)	22.83(1.57)	83.79(1.67)	32.5(7.45)	44.25(7.33)	25.98(7.15)	0.44(0.1)	9.95(2.28)
			ARIMA	25(0)	22.13(1.58)	84.36(1.77)	70.5(3.69)	61.04(4.33)	83.64(3.89)	0.73(0.13)	10.18(0.94)
	Individual order		AR	25(0)	22.97(2.37)	83.4(3.17)	28.96(12.78)	42.07(11.36)	23.05(12.16)	0.39(0.12)	8.64(4.93)
		30 min	ARMA	25(0)	23.04(2.22)	82.7(3.3)	24.25(11.17)	37.49(11.5)	18.85(10.4)	0.4(0.13)	9.55(4.77)
		specific	ARIMA	25(0)	22.45(1.29)	84.26(1.81)	66.63(5.43)	60.54(6.63)	74.08(6.94)	0.58(0.17)	10(0)
	Individual order	Day and night	AR	25(0)	24.15(1.53)	78.87(1.83)	27.12(4.38)	37.31(7.97)	21.31(3.14)	0.32(0.07)	10(4.11)
			ARMA	25(0)	24.44(1.59)	78.75(2.18)	25.57(4.96)	36.95(10.63)	19.55(3.39)	0.28(0.08)	9(4.04)
			ARIMA	25(0)	22.93(1.31)	83.68(1.56)	66.37(5.09)	68.36(5.64)	64.98(7.33)	0.41(0.13)	9.86(0.75)
	Regularized		AR	21.82(2.43)	22.87(1.63)	83.1(2.11)	43.53(6.27)	48.5(5.59)	39.72(7.2)	0.57(0.1)	11.36(2.54)
	RLS		AR	29.82(0.94)	27.67(1.6)	76.12(2.11)	63.89(4.46)	51.43(5)	84.32(5.16)	1.01(0.15)	16.36(2.4)
	Population		SVR	24.45(2.99)	22.72(2.75)	81.69(8.39)	50.81(13.21)	47.59(11.73)	44.15(12.83)	0.56(0.33)	9.79(3.03)
			RegRF	25.09(0.67)	23.35(1.77)	80.91(1.89)	19.57(11.74)	23.99(16.56)	12.24(11.07)	0.43(0.18)	8.47(4.22)
			fNN	21.36(2.25)	21.74(1.45)	85.93(1.7)	26.15(10.79)	37.6(11.79)	20.58(9.98)	0.43(0.14)	6.91(3.57)
			LSTM	24.55(1.45)	22.97(1.99)	83.25(2.28)	20.52(13.13)	40.6(15.03)	15.03(12.46)	0.28(0.15)	8.32(6.16)
Individual			SVR	24.27(3.52)	22.6(4.62)	82.89(11.77)	53.82(12.75)	59.91(11.02)	49.54(12.67)	0.47(0.35)	11.15(3.26)
	Population		RegRF	25.55(1.57)	23.38(2.02)	78.23(2.06)	31.37(9.65)	47.11(8.59)	24.42(9.5)	0.36(0.12)	11.91(3.04)
	hyperparameters		fNN	20.18(0.94)	21.78(1.78)	84.78(1.49)	38.58(7.56)	47.95(7.54)	32.89(8.47)	0.48(0.14)	10.59(2.15)
			SVR	23.64(2.25)	22.21(2.09)	81.32(2.36)	53.63(7.86)	58.21(7.16)	49.71(9.69)	0.55(0.13)	12.36(2.82)
	Individual		RegRF	25(0)	26.06(2.02)	77(2.31)	40.34(6.68)	50.81(7.04)	33.93(7.24)	0.44(0.11)	15.36(3.38)
	hyperparameters		fNN	20.09(0.67)	21.63(1.69)	85.1(1.45)	37.54(7.59)	47.02(8)	31.77(8.14)	0.49(0.13)	10.14(2.12)
		Day and night	SVR	24(2.02)	29.22(2.33)	71.35(4.33)	45.85(6.85)	53.92(7.98)	39.89(7.04)	0.52(0.12)	12.55(3.25)
	Individual		RegRF	25(0)	29.72(2.06)	69.69(3.2)	34.49(6.11)	45.47(6.39)	28.28(6.7)	0.45(0.1)	14.41(3.91)
	hyperparameters		fNN	20.73(1.78)	23.54(1.91)	82.11(1.92)	32.25(6.88)	48.69(7.4)	24.5(6.56)	0.35(0.1)	11.18(3.22)

## Data Availability

Not applicable.
